# MORO: a Cytoscape app for relationship analysis between modularity and robustness in large-scale biological networks

**DOI:** 10.1186/s12918-016-0363-3

**Published:** 2016-12-23

**Authors:** Cong-Doan Truong, Tien-Dzung Tran, Yung-Keun Kwon

**Affiliations:** 1 Department of IT Convergence, University of Ulsan, 93 Daehak-ro, Nam-gu, Ulsan, 680-749 Republic of Korea; 2 Complex Network and Bioinformatics Group, Center for Research and Development, Hanoi University of Industry, Hanoi, Vietnam

**Keywords:** Cytoscape app, Boolean network, Network robustness, Modularity, Centrality, Gene-ontology, Parallel computation, OpenCL

## Abstract

**Background:**

Although there have been many studies revealing that dynamic robustness of a biological network is related to its modularity characteristics, no proper tool exists to investigate the relation between network dynamics and modularity.

**Results:**

Accordingly, we developed a novel Cytoscape app called MORO, which can conveniently analyze the relationship between network modularity and robustness. We employed an existing algorithm to analyze the modularity of directed graphs and a Boolean network model for robustness calculation. In particular, to ensure the robustness algorithm’s applicability to large-scale networks, we implemented it as a parallel algorithm by using the OpenCL library. A batch-mode simulation function was also developed to verify whether an observed relationship between modularity and robustness is conserved in a large set of randomly structured networks. The app provides various visualization modes to better elucidate topological relations between modules, and tabular results of centrality and gene ontology enrichment analyses of modules. We tested the proposed app to analyze large signaling networks and showed an interesting relationship between network modularity and robustness.

**Conclusions:**

Our app can be a promising tool which efficiently analyzes the relationship between modularity and robustness in large signaling networks.

**Electronic supplementary material:**

The online version of this article (doi:10.1186/s12918-016-0363-3) contains supplementary material, which is available to authorized users.

## Background

*Network modularity* represents the degree to which a network is divided into modules of separate community structures. A highly modularized network has dense connectivity between the nodes within each module but sparse connectivity between the nodes of different modules. Many plugins based on the Cytoscape platform [1] have been developed for modularity analysis in biological networks. For example, clusterMaker [2] implemented several clustering algorithms such as k-means, k-medoid, SCPS, and AutoSOME to visualize a structure of modules within biological networks. GIANT [3] was proposed to investigate topological or functional relationships in a metabolic network by performing a clustering analysis and a functional cartography of nodes. Another well-known plugin is NeMo [4], which can identify diverse network communities by means of a neighbor-sharing score based on a hierarchical agglomerative clustering method. These plugins have a limitation, though, in that they focus only on the structural analysis of a network and its visualization, without any consideration of dynamics analysis. This restricts their use to undirected networks such as protein–protein networks, or to analysis of directed networks that ignores the direction information.

Herein we note previous studies showing that dynamical behaviors, particularly robustness, of biological networks can be highly affected by their modularity characteristics. For instance, a recent study reported that a modular organization of cancer signaling networks is associated with the patient survivability, which suggests a relationship between modularity and network robustness [5]. Also, the robustness against state perturbations of a human signaling network was negatively correlated to network modularity [6]. Modular stabilizing in protein–protein interaction networks can be recombined to create highly robust chimeric proteins in evolution [7]. It has been also argued that modularity reduces robustness against mutation in metabolic networks [8]. Because of the importance of network modularity and robustness, there is a pressing need to develop a tool that can analyze both simultaneously. Accordingly, we devised a novel Cytoscape app called MORO that can analyze a relationship between dynamical robustness and structural modularity in biological networks represented by directed graphs. In addition, to make it possible to analyze very large-scale networks, we implemented the robustness computation portion of the app as a parallel algorithm by using the OpenCL library. It was also designed to efficiently visualize how the detected modules are located relative to each other. Furthermore, it elucidates analysis results of centrality and gene ontology (GO) enrichment of modules. Moreover, it provides a batch-mode simulation function to validate whether a result observed in a biological network is consistently conserved in many randomly organized networks. In this study, we tested our app in a case study investigating large-scale signaling networks and observed that modularity and robustness are negatively correlated, similar to previous findings [6]. It was verified by means of batch-mode simulation that these findings hold in random networks. Moreover, we found some GO terms which are differently enriched between the largest module and the rest of the modules, and it was shown that the module size is positively correlated with five centrality values. In summary, our app can efficiently analyze the relationship between modularity and robustness in large signaling networks.

## Methods

**Fig. 1:**
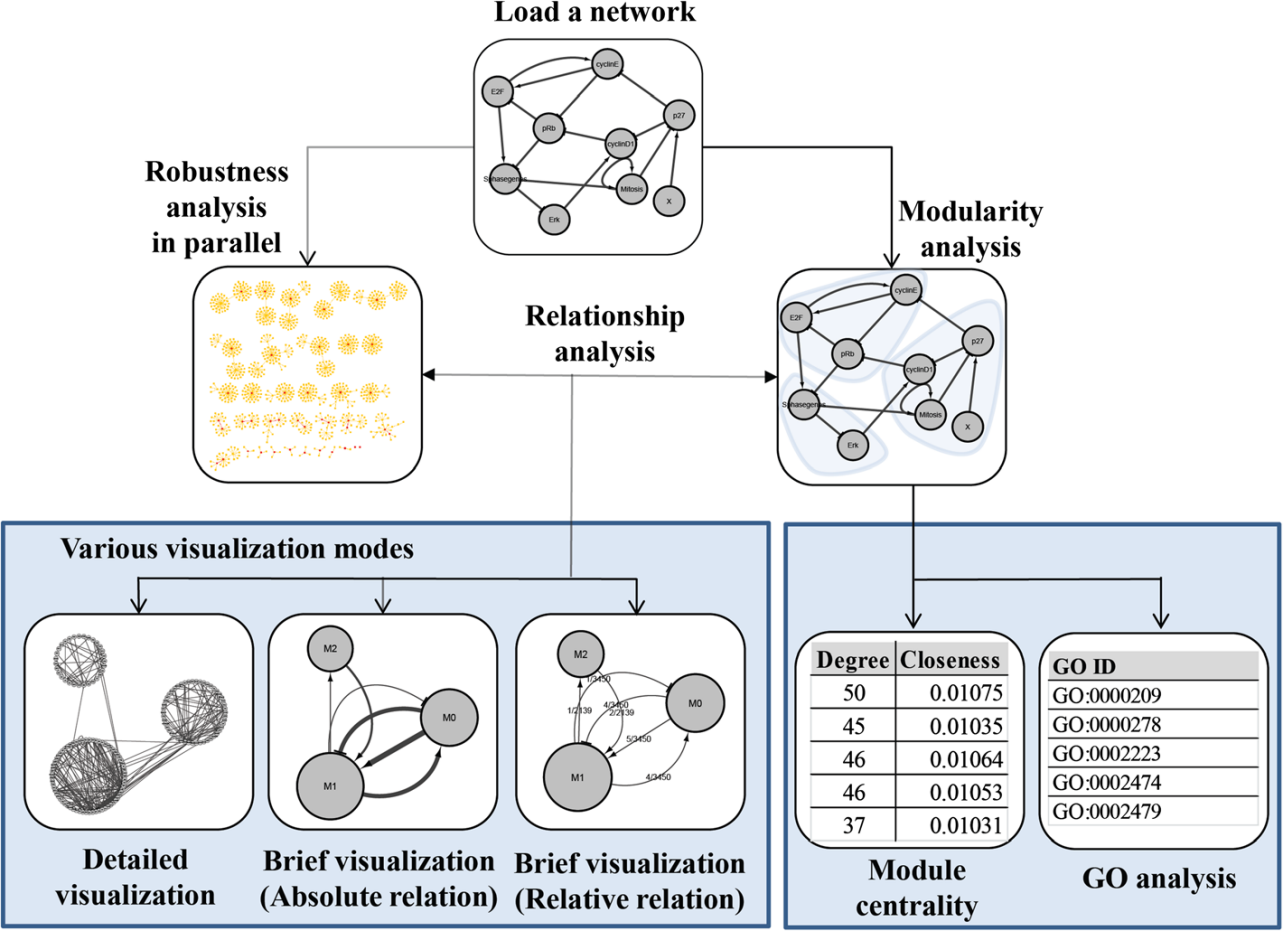
The overall process to analyze the relationship between the network robustness and modularity in MORO. After a directed network is loaded for analysis, the network modularity and robustness are calculated. In particular, the time consuming part is processed in parallel by using multi-core CPU or GPU. The analysis result can be checked by three types of visualization modes and a summary table. The centrality values and GO analysis of modules are additionally provided

Figure 1 illustrates the main process of our app. Firstly, a directed network is loaded for analysis. Next, the app computes the modularity and robustness of the network. In particular, the robustness algorithm was implemented in parallel computation by using the OpenCL library. The results can be visualized in three modes: a detailed visualization mode, a brief visualization with absolute relations, and a brief visualization with relative relations. Also, the results can be summarized in tables that include centrality and gene ontology analyses. Details of this process are given in the following subsections.

### Network modularity

Given a network represented by a directed graph *G*(*V*, *A*) where *V* and *A* are the sets of nodes and interactions, respectively, we employ the modularity measure introduced in a previous study [9]. A partition *P* = {*V*_1_, *V*_2_, …, *V*_*M*_} of *V* is a set of nonempty disjoint subsets of *V* that covers *V* (i.e. *V*_*i*_ ∩ *V*_*j*_ = ∅ for all *i*, *j* ∈ {1, 2, …, *M*} and *i* ≠ *j*, and U_*i* = 1_^*M*^ *V*_*i*_ = *V*). Then, the modularity of the partition *M*(*P*) is defined as $M(P)={\displaystyle {\sum}_{i=1}^M\left(\frac{\omega_{V_i{V}_i}}{\omega }-\frac{\omega_{V_i}^{in}{\omega}_{V_i}^{out}}{\omega^2}\right)}$, where ${\omega}_{V_i{V}_i}$ is the number of interactions whose starting and ending nodes are both included in module *V*_*i*_, ${\omega}_{V_i}^{out}$, and ${\omega}_{V_i}^{in}$ are the numbers of interactions whose starting or ending nodes only, respectively, are included in module *V*_*i*_, and *ω* is the total number of interactions in the network. Then, the modularity of the network is defined as *M*(*G*) = *max*_*P*_ *M*(*P*). However, it is difficult to obtain the optimal partition. In our app, the modularity value of a network is averaged over 30 trials by using an optimization algorithm proposed in a previous study [10].

### Robustness dynamics in a Boolean network model

A Boolean network model has been used to examine robustness-related dynamics of signaling networks and has been employed to investigate the dynamics of various biological networks [11–17]. A Boolean network is represented by a directed graph *G*(*V*, *A*) where *V* = {*v*_1_, *v*_2_, …, *v*_*N*_} is a set of Boolean variables and *A* is a set of ordered pairs of Boolean variables called directed links. Each *v*_*i*_ ∈ *V* has a value of 1 (‘on’) or 0 (‘off’) that represents the state of the corresponding element. A directed link (*v*_*i*_, *v*_*j*_) has a positive (‘activating’) or negative (‘inhibiting’) relationship from *v*_*i*_ to *v*_*j*_. In this model, each state *s*(*t*) = (*v*_1_(*t*), *v*_2_(*t*), …, *v*_*N*_(*t*)) at time *t* transits to the next state *s*(*t* + 1) according to the set of update rules *F* = {*f*_1_, *f*_2_, …, *f*_*N*_}, i.e., *s*(*t* + 1) = *F*(*s*(*t*)), where we randomly choose either a logical conjunction or disjunction for *f*_*i*_ with a uniform probability distribution. For instance, if a Boolean variable *v* has a positive relationship from *v*_1_, a negative relationship from *v*_2_ and a positive relationship from *v*_3_, then the conjunction and disjunction update rules are $v\left(t+1\right)={v}_1(t)\wedge {\overline{v}}_2(t)\wedge {v}_3(t)$ and $v\left(t+1\right)={v}_1(t)\vee {\overline{v}}_2(t)\vee {v}_3(t)$, respectively. In the case of the conjunction, the value of *v* at time *t* + 1 is 1 only if the values of *v*_1_, *v*_2_ and *v*_3_ at time *t* are 1, 0 and 1, respectively. A state of *G* is defined as a vector of values *v*_1_ through *v*_*N*_. A state trajectory starts from an initial state *s*(0) and eventually converges to either a fixed-point or limit-cycle attractor. Because these attractors can represent diverse biological network behaviors such as multistability, homeostasis, and oscillation, a change in the converging attractor can be interpreted as a loss of robustness. We denote the attractor converged to starting from an initial state *s*(0) by 〈*s*〉. The network is considered to be robust against mutation at *v*_*i*_ if 〈*s*〉 is equal to $\left\langle {s}_{{\overline{v}}_i}\right\rangle$, where ${\overline{v}}_i\left(=\neg {v}_i\right)$ indicates the state perturbation of *s* subjected to *v*_*i*_. This concept to measure robustness has been widely used [18–20]. More specifically, the robustness of a network *γ*(*G*) is defined as follows:

$$ \gamma (G)=\frac{1}{N\cdot \left|S\right|}{\displaystyle \sum_{s\in S}}{\displaystyle \sum_{i=1}^N}I\left(\left\langle s\right\rangle =\left\langle {s}_{{\overline{v}}_i}\right\rangle \right), $$

where *S* is the set of whole states (i.e. *S* = 2^*N*^), and *I*(⋅) is an indicator function. Because |*S*| is a very large number, we used a sample subset $\tilde{S}\subseteq S$ with $\left|\tilde{S}\right|=2N$ instead of *S* to calculate *γ*(*G*). Given a partition *P* = {*V*_1_, *V*_2_, …, *V*_*M*_}, we employed the in-module and out-module robustness of a module *V*_*i*_, *γ*_*in*_(*V*_*i*_), and *γ*_*out*_(*V*_*i*_), respectively, defined in [6] as follows:

$$ {\gamma}_{in}\left({V}_i\right)=\frac{1}{\left|\tilde{S}\right|}{\displaystyle \sum_{s\in \tilde{S}}}{\displaystyle \sum_{v\in {V}_i}}\frac{H\left({{\displaystyle \prod}}_{V_i}\left\langle s\right\rangle, \kern0.75em {{\displaystyle \prod}}_{V_i}\left\langle {s}_{\overline{v}}\right\rangle \right)}{\left|{V}_i\right|} $$

and

$$ {\gamma}_{out}\left({V}_i\right)=\frac{1}{\left|\tilde{S}\right|}{\displaystyle \sum_{s\in \tilde{S}}}{\displaystyle \sum_{v\in {V}_i}}\frac{H\left({{\displaystyle \prod}}_{V\backslash {V}_i}\left\langle s\right\rangle, \kern0.75em {{\displaystyle \prod}}_{V\backslash {V}_i}\left\langle {s}_{\overline{v}}\right\rangle \right)}{\left|{V}_i\right|}, $$

where ${{\displaystyle \prod}}_{V_i}\left\langle s\right\rangle$ represents a projection operator to extract the partial attractor of a given subset *V*_*i*_ ⊆ *V* from an attractor 〈*s*〉, and *H*(〈*s*〉, 〈*s* ′ 〉) denotes a similarity measure between two attractors 〈*s*〉 and 〈*s* ′ 〉. More particularly, given 〈*s*〉 = *s*_0_ → *s*_1_ → … → *s*_*l* − 1_ and $\left\langle {s}^{\prime}\right\rangle ={s}_0^{\prime}\to {s}_1^{\prime}\to \dots \to {s}_{l^{\prime }-1}^{\prime }$ (1 ≤ *l* ≤ *l*′ is assumed without loss of generality), *H*(〈*s*〉, 〈*s*′〉) is defined as follows:

$$ H\left(\left\langle s\right\rangle, \left\langle {s}^{\prime}\right\rangle \right)=\frac{1}{l^{\prime }}{\displaystyle \sum_{j=0}^{l-1}}\left(1-\frac{h\left({s}_j,{s}_j^{\prime}\right)}{N}\right) $$

where *h* is the Hamming distance (i.e. the number of different bits between two binary sequences). Then, the in-module and out-module robustness of a network, *γ*_*in*_(*V*_*i*_) and *γ*_*out*_(*V*_*i*_), respectively, are defined as follows:

$$ {\gamma}_{in}(G)=\frac{1}{M}{\displaystyle \sum_{i=1}^M}{\gamma}_{in}\left({V}_i\right) $$

and

$$ {\gamma}_{out}(G)=\frac{1}{M}{\displaystyle \sum_{i=1}^M}{\gamma}_{out}\left({V}_i\right) $$

### Parallel computation of robustness

In our app, we employed a Boolean network model to compute robustness. In particular, we further calculated in-module and out-module robustness which represent how much the module subject to a perturbation and the groups of other modules, respectively, are robust against the perturbation. Unfortunately, it is very time-consuming to compute robustness. To reduce the running time, we implemented the robustness calculation part of the app as a parallel algorithm by using the OpenCL library (see Additional file 1: Text S1).

### A batch-mode simulation on random Boolean networks

We developed a function for a batch-mode simulation on random Boolean networks (RBNs) to examine if a finding in biological networks holds in RBNs or not similarly in a previous study [12, 19, 21–26]. The batch-mode simulation requires two steps for configuring parameters. The first step is to select an RBN generation model from among five models: Barabási-Albert (BA) model [27], Erdős-Rényi (ER) model [28], an Erdős-Rényi variant model [29] and two shuffling models [23, 30, 31], and the second step is to set the number of considered initial-states and the type of update-rule schemes (see the subsection “Robustness dynamics in a Boolean network model” for details). Once computations of modularity and robustness are completed, all results are saved in a resulting file, “net_based_result.txt” which describes modularity and robustness results of each RBN (see Additional file 1: Text S2).

### Visualization of relations between modules

Our app provides three types of visualizations to show the relationship between modules. The first type is a detailed visualization mode in which all nodes and interactions of the loaded network are shown and the nodes are grouped into modules placed by using the Cytoscape group attributes layout. The second type is a brief visualization mode with absolute relations, in which a group node corresponds to a detected module and the weight of a link between group nodes denotes the number of interactions between a pair of modules. The last mode is the same as the second mode except that the weight of a link denotes the ratio of the number of interactions between a pair of modules to the maximal possible number of interactions between them, that is *w*/(*n*_1_ *n*_2_), where *w* is the number of actual interactions between the pair of modules, and *n*_1_ and *n*_2_ are the numbers of nodes included in each of the modules.

### Module centrality and GO analyses

Many previous studies have shown that the centrality properties of genes/proteins in biological networks are strongly related to their functional roles in a topological or dynamical sense. To extend this concept to module-based centrality analysis, we implemented a function to examine five centrality measures including degree [32], closeness [33], betweenness [34], stress [35] and eigenvector [36] of modules (see Additional file 1: Text S3). Besides, we developed a GO analysis function to compare the functional difference between two groups of modules. To this end, we first identify two groups of genes by selecting some modules of interest. Then, Entrez gene id is mapped to UniProtKB by utilizing the web service at http://www.uniprot.org/ [37], and all relevant GO terms are extracted by using the web service at http://www.ebi.ac.uk/QuickGO/ [38]. Finally, GO terms which are most differently enriched between the two gene groups are listed in a table or exported into a text file.

## Results and discussion

In this section, we tested MORO with two large-scale signalling networks, the canonical cell signaling network (STKE; http://stke.sciencemag.org) and the human signal transduction network (HSN; http://www.bri.nrc.ca/wang) which consist of 754 proteins and 1624 interactions, and 5443 genes and 37,663 interactions, respectively.

### Analysis of modularity and robustness

**Fig. 2:**
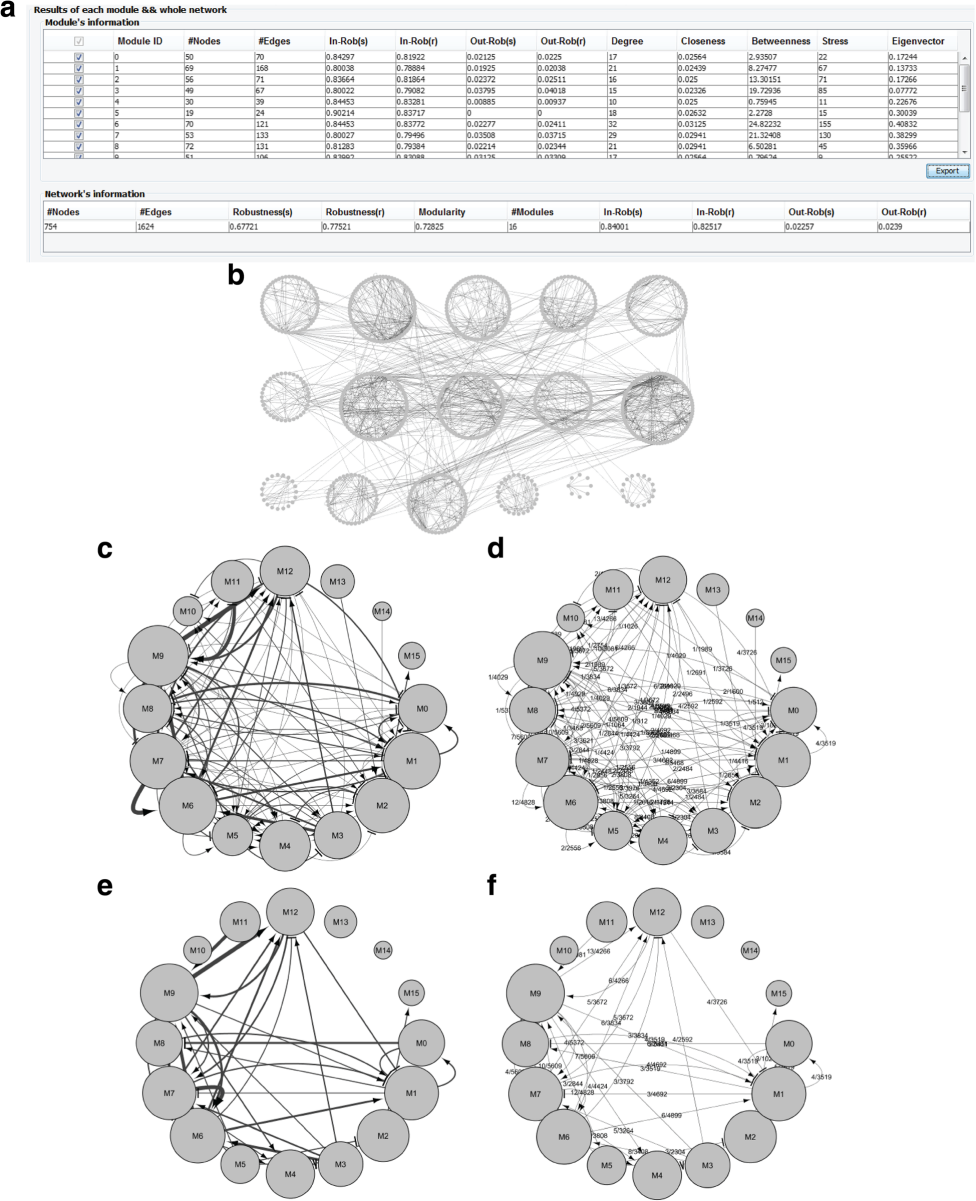
Analysis results of the STKE network by MORO. **a** A summary table. Modularity and robustness results in module and network levels are listed in the upper and the lower tables, respectively. **b** Result of the detailed visualization mode. We found a total of 16 modules each of which is represented by a circular list of genes. **c**-**d** Results of the brief visualization mode with absolute and relative relations, respectively. Each module is represented by a single group node whose radius is proportional to the number of nodes belonging to the module. The weight of a link denotes the number of interactions between the corresponding pair of modules and the ratio of the number of interactions between a pair of modules to the maximal possible number of interactions between them in (**c**) and (**d**), respectively. **e**-**f** The reduced visualization results. They are subnetworks induced from (**c**) and (**d**), respectively, by removing all links except about 30% of links with the highest weight values (This is performed by specifying the appearance ratio parameter in MORO)

The analysis and visualization results of the STKE and HSN networks are shown in Fig. 2 and Additional file 2: Figure S1, respectively. In particular, Fig. 2(a) and in Additional file 2: Figure S1(a) explain various summarized results including the number of modules, modularity, robustness, in-/out-module robustness, and centrality values. Specifically, the number of modules were 16 and 22, the modularity values were 0.72825 and 0.54534, and the robustness values were 0.67721 and 0.75400 in the STKE and HSN networks, respectively. By selecting the visualization option, we can observe the relation between the detected modules in three different modes: a detailed mode (Fig. 2(b) and in Additional file 2: Figure S1(b)), a brief mode with absolute relations (Fig. 2(c) and in Additional file 2: Figure S1(c)), and a brief mode with relative relations (Fig. 2(d) and in Additional file 2: Figure S1(d)). In the detailed mode, each module is represented by a circular group of genes and all interactions between the genes are presented in the network. In other words, the visualized network is actually same with the first given network except that the genes belonging to a same module are located close to each other. On the other hand, each module is represented by a single node and a relation between modules is represented by a directed link in both of the brief modes. The only difference between the two brief modes is that the weight of a link means the number of interactions between a pair of modules in the brief mode with absolute relations, whereas it means the ratio of the number of interactions between a pair of modules to the maximal possible number of interactions between them. By properly specifying the appearance ratio parameter which is defined the ratio of the number of interactions to be visible over the total number of interactions between modules, we can retrieve more reduced information about the brief relations between modules. For example about the STKE network, Fig. 2(e) and (f) shows the visualization results reduced from Fig. 2(c) and (d), respectively, by specifying the appearance ratio to 0.3. Then, we can identified which module is strongly interacting with or isolated from other modules (see Additional file 2: Figure S1(e) and (f) for the result of the HSN network).

To validate effectiveness of our app, we also conducted the same case study about large-scale signaling networks as in a previous study [6] which reported that the network modularity tends to be negatively correlated to the robustness against state perturbations. To reproduce such a negative relationship between network modularity and the robustness in this study, we generated 6400 random Boolean networks and computed the robustness and the modularity of each network by using MORO. We note that this extensive simulation could be conducted in a practical time by the parallel implementation of main functions in MORO. As a result, we could observe the same negative relationship between the modularity and the robustness, consistent to the result in [6] (see Additional file 2: Figure S2(a)). In addition, we observed that the results of STKE and HSN are very close to the trend line of the random Boolean networks. Moreover, we could also observe that the in-module robustness is clearly negatively correlated with the network modularity (Additional file 2: Figure S2(b)), whereas the out-module robustness is not (Additional file 2: Figure S2(c)). In addition, the in-module robustness was positively correlated with the network robustness (Additional file 2: Figure S2(d)), whereas the out-module robustness was not (Additional file 2: Figure S2(e)). As explained in the previous study, we could also conclude that the negative relationship between network robustness and modularity is mainly caused by the relationship between in-module robustness and network modularity through intensive simulations using our app.

### Time performance analysis

To show the computational cost of MORO, we examined the running time in calculating robustness and modularity in the HSN and STKE networks. We tested the app on a system with an NVIDIA GeForece GTX 680 GPU with 1536 processor at 1GHz, seven-core Intel(R) Core i7-4770 K CPU 3.50 GHz, and 16 GB of memory. Table 1 shows the result. In case of the HSN network, the speedup by the GPU-based parallel computation over the single-CPU was slightly greater than that by multi-core CPU, and both speedups were proportional to the number of considered initial states. On the other hand, it is interesting that the speedup by multi-core CPU was greater than that by GPU, and both were not proportional to the number of initial states in case of the STKE network. We infer that the analysis of the STKE network was terminated before the parallel computation power is fully utilized due to the relatively small size of the network. Taken together, we can efficiently analyze the relation between robustness and modularity in large-scale networks by parallel computation with two options, multi-core CPU and GPU.

**Table 1:** Running time of MORO

Number of considered initial-states (*S*)	Single CPU (A)	Multi-core CPU (B)	Speedup (A/B)	GPU (C)	Speedup (A/C)
HSN network					
50	467:00:15	00:10:13	2744	00:09:58	2925
100	934:52:07	00:20:01	5488	00:19:16	5850
150	1401:47:01	00:30:39	8232	00:28:75	8775
200	1869:03:03	00:40:52	10976	00:38:38	11700
1000	9345:16:06	03:24:33	54880	03:11:01	58500
STKE network					
50	01:22:50	00:00:06	825	00:00:13	380
100	02:45:00	00:00:10	990	00:00:24	412
150	04:07:15	00:00:14	1060	00:00:35	424
200	05:30:00	00:00:18	1100	00:00:46	430
1000	27:30:00	00:01:27	1137	00:03:40	450

A total of running time to compute robustness and modularity is compared among single-CPU, multi-core CPU, and GPU options. Time is formatted as *hh:mm:ss*

### Module centrality analysis

**Fig. 3:**
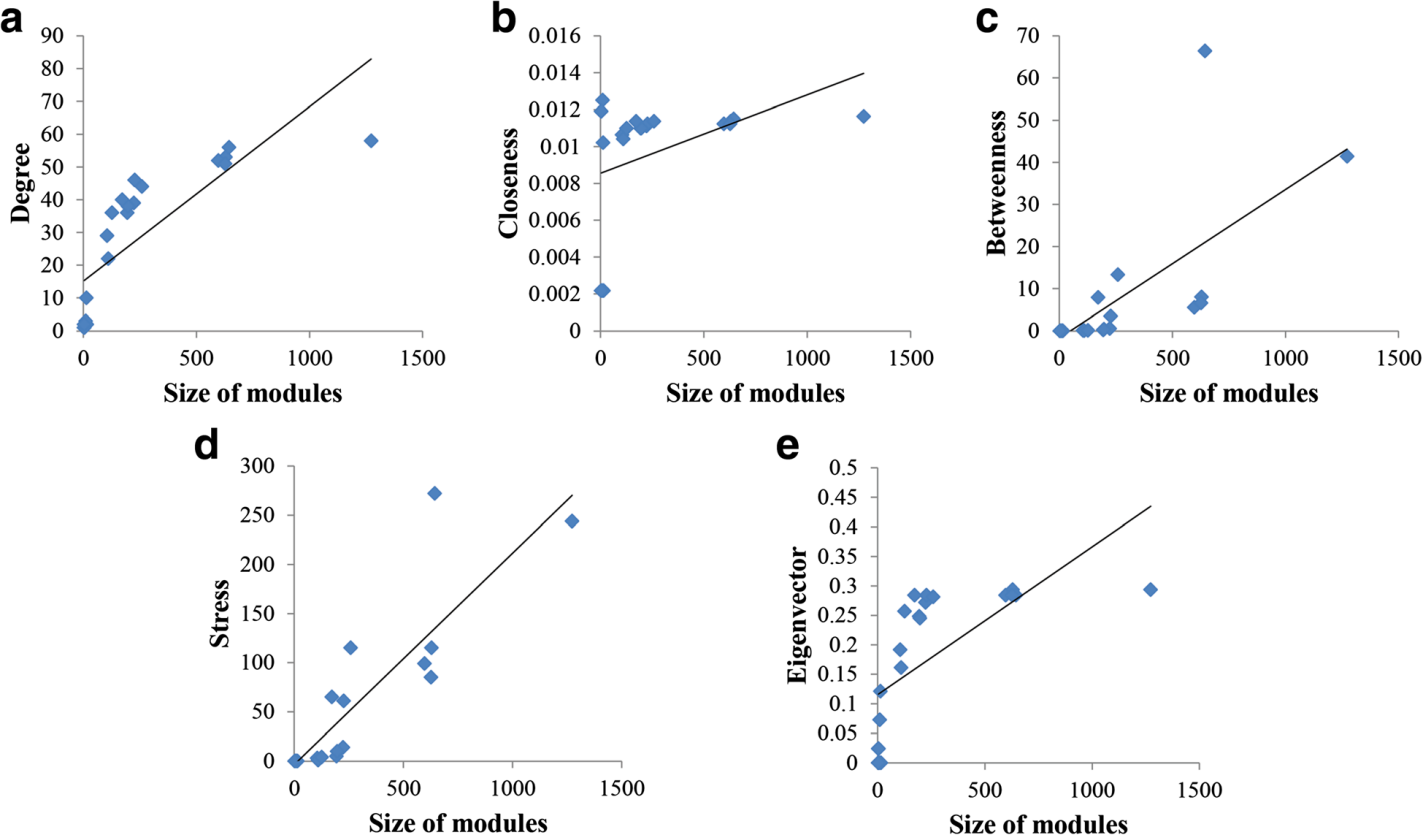
Changes of module centrality values against the module size in the STKE network. **a-e** Results with respect to degree, closeness, betweenness, stress, and eigenvalue. The module size which is defined as the number of nodes belonging to the module showed positive relationships with all module centrality measures. The correlation coefficients in (**a**)-(**e**) were 0.75339, 0.564168, 0.599553, 0.657316, and 0.511411, respectively, with all p-value < 10^−4^

After we obtain the modular structure of a network, we can analyse the centrality of modules based on the brief mode visualization result. Specifically, we consider a module network where a node and a link represent a module and the set of interactions between a pair of modules, respectively. Then, we can examine five well-known centrality values such as degree, closeness, betweenness, stress, and eigenvector in the module network. In this case study, we examined the change of the centrality values against the module size, which is defined by the number genes belonging to a module, in the STKE (Fig. 3) and HSN (Additional file 2: Figure S3) networks. It is interesting that all centrality measures or all except closeness showed the positive relations with the module size in the STKE and HSN networks, respectively. In other words, the module was likely to be more central as the module size gets larger. To investigate if this property is reserved in random networks, we generated two groups of 100 random networks by shuffling interactions of the STKE and HSN networks while preserving a degree distribution, and examined the change of the centrality values against the module size (see Additional file 2: Figures S4 and S5). Similar to the result in the signaling networks, the module size was positively correlated with the centrality values in the random networks. This suggests that the hub modules might play an important role in the community network [39–41]. Additionally, we examined the relationship between the in-/out-module robustness and the module centrality values in the STKE and HSN networks (see Additional file 2: Figures S6 and S7). Unlike the relation with the module size, the in-/out-module robustness was not significantly correlated with the centrality values. In other words, the centrality of modules cannot indicate the in-/out-module robustness in the signaling network.

### GO analysis

It is possible to analyze GO enrichment [42] by using MORO. To show this function, we first specified two groups of genes, which consist of the genes in the largest module (1042 genes) and the rest of genes (4401 genes), respectively, in the HSN network. Table 2 shows all GO terms which were more enriched in the largest module than in the others: cytoplasm, nucleus, and protein complex in cellular component terms; protein, metal ion, nucleotide, and DNA bindings in molecular function terms; gene expression, viral process, and regulation of DNA-templated transcription in biological processes terms. As a result, MORO can provide the useful information about GO analysis between any two groups of modules.

**Table 2:** GO analysis in the HSN network

Category	GO Terms	The largest module		The rest of genes		*P*-value
		No. of genes %		No. of genes %		
Cellular component	Cytoplasm	161	20.33	767	16.49	0.00794
	Nucleus	230	29.04	531	11.42	0
	Protein complex	26	3.28	83	1.79	0.0054
Molecular Function	Protein binding	249	31.44	1115	23.97	0.00001
	Metal ion binding	85	10.73	351	7.55	0.00227
	Nucleotide binding	58	7.32	234	5.03	0.00814
	DNA binding	127	16.04	150	3.23	0
Biological Process	Gene expression	40	5.05	139	2.99	0.00263
	Viral process	38	4.80	132	2.84	0.00338
	Regulation of transcription, DNA-templated	115	14.52	177	3.81	0

GO terms which are significantly enriched between the largest module and the rest of modules are listed. All *P*-values are calculated by using a z-test

## Conclusions

Many recent reports have reported that robust behavior against mutations might be correlated to the modularity of a signaling network. Motivated by these results, we developed a novel Cytoscape app called MORO, which can analyze the relationship between network robustness and modularity. We implemented it in parallel by using the OpenCL library to allow application to very-large-scale networks. In addition, our app can provide information about topological relations between modules by means of various visualization modes and centrality analysis. MORO includes also five centrality measures which can examine how centrally each module is positioned in terms of relations among the modules. Moreover, it can conveniently analyze the gene ontology enrichment of modules only if Entrez id of gene is given. A batch-mode simulation function was also included to allow verification of whether a finding is a design principle of random networks. In the future, MORO will be extended to consider various types of mutations such as a knockout and edge mutation, and to analyze publicly-available signaling networks represented by ordinary differential equations by devising a conversion method from continuous models to Boolean networks.

## Additional files

Additional file 1: Text S1. Parallel robustness computation based on the OpenCL. **Text S2.** Output file by the batch-mode simulation on RBNs. **Text S3.** Centrality measures. (PDF 753 kb)
Additional file 2: Figure S1. Analysis results of the HSN network by MORO. **Figure S2.** Correlations between the modularity and robustness of 6,400 random Boolean networks where the number of nodes is 50 and the number of interactions is in the range of [49, 2031]. **Figure S3.** Changes of module centrality values against the module size in the HSN network. **Figure S4.** Changes of module centrality values against the module size in STKE-shuffled random networks. **Figure S5.** Changes of module centrality values against the module size in HSN-shuffled random networks. **Figure S6.** Correlation between module centrality values and in-/out-module robustness in the STKE network. **Figure S7.** Correlation between module centrality values and in/out-module robustness in the HSN network. (PDF 1052 kb)

## Abbreviations

GO: Gene ontology
MORO: Cytoscape app for modularity and robustness analysis
RBN: Random Boolean network

## References

[1] Shannon P, Markiel A, Ozier O, Baliga NS, Wang JT, Ramage D, Amin N, Schwikowski B, Ideker T (2003). Cytoscape: a software environment for integrated models of biomolecular interaction networks. Genome Res.

[2] Morris J, Apeltsin L, Newman A, Baumbach J, Wittkop T, Su G, Bader G, Ferrin T (2011). clusterMaker: a multi-algorithm clustering plugin for Cytoscape. BMC Bioinformatics.

[3] Cumbo F, Paci P, Santoni D, Di Paola L, Giuliani A (2014). GIANT: a cytoscape plugin for modular networks. PLoS One.

[4] Rivera C, Vakil R, Bader J (2010). NeMo: Network Module identification in Cytoscape. BMC Bioinformatics.

[5] Takemoto K, Kihara K (2013). Modular organization of cancer signaling networks is associated with patient survivability. Biosystems.

[6] Tran T-D, Kwon Y-K (2013). The relationship between modularity and robustness in signalling networks. J R Soc Interface.

[7] Lin Y-S, Hsu W-L, Hwang J-K, Li W-H (2007). Proportion of solvent-exposed amino acids in a protein and rate of protein evolution. Mol Biol Evol.

[8] Holme P (2011). Metabolic robustness and network modularity: a model study. PLoS One.

[9] Leicht EA, Newman MEJ (2008). Community structure in directed networks. Phys Rev Lett.

[10] Noack A (2009). Modularity clustering is force-directed layout. Physical Review E.

[11] Kauffman S, Peterson C, Samuelsson B, Troein C (2003). Random Boolean network models and the yeast transcriptional network. Proc Natl Acad Sci.

[12] Kwon Y-K, Choi S, Cho K-H (2007). Investigations into the relationship between feedback loops and functional importance of a signal transduction network based on Boolean network modeling. BMC Bioinformatics.

[13] Shmulevich I, Lähdesmäki H, Dougherty ER, Astola J, Zhang W (2003). The role of certain Post classes in Boolean network models of genetic networks. Proc Natl Acad Sci.

[14] Kwon YK, Kim J, Cho KH. Dynamical Robustness Against Multiple Mutations in Signaling Networks. IEEE/ACM Trans Comput Biol Bioinform. 2016;13(5):996-1002. 10.1109/TCBB.2015.2495251 26529781

[15] Kwon Y-K, Cho K-H (2007). Boolean dynamics of biological networks with multiple coupled feedback loops. Biophys J.

[16] Fauré A, Naldi A, Chaouiya C, Thieffry D (2006). Dynamical analysis of a generic Boolean model for the control of the mammalian cell cycle. Bioinformatics.

[17] Garg A, Mohanram K, Di Cara A, De Micheli G, Xenarios I (2009). Modeling stochasticity and robustness in gene regulatory networks. Bioinformatics.

[18] Ciliberti S, Martin OC, Wagner A (2007). Robustness can evolve gradually in complex regulatory gene networks with varying topology. PLoS Comput Biol.

[19] Kwon Y-K, Cho K-H (2008). Quantitative analysis of robustness and fragility in biological networks based on feedback dynamics. Bioinformatics.

[20] Kitano H (2004). Biological robustness. Nat Rev Genet.

[21] Kwon Y-K, Cho K-H (2008). Coherent coupling of feedback loops: a design principle of cell signaling networks. Bioinformatics.

[22] Le D-H, Kwon Y-K (2011). The effects of feedback loops on disease comorbidity in human signaling networks. Bioinformatics.

[23] Le D-H, Kwon Y-K (2013). A coherent feedforward loop design principle to sustain robustness of biological networks. Bioinformatics.

[24] Trinh H-C, Le D-H, Kwon Y-K (2014). PANET: a GPU-based tool for fast parallel analysis of robustness dynamics and feed-forward/feedback loop structures in large-scale biological networks. PLoS One.

[25] Trinh H-C, Kwon Y-K (2015). Effective Boolean dynamics analysis to identify functionally important genes in large-scale signaling networks. Biosystems.

[26] Campbell C, Albert R (2014). Stabilization of perturbed Boolean network attractors through compensatory interactions. BMC Syst Biol.

[27] Barabási A-L, Albert R (1999). Emergence of Scaling in Random Networks. Science.

[28] Erdős P, Rényi A (1959). On random graphs, I. Publicationes Mathematicae (Debrecen).

[29] Le D-H, Kwon Y-K (2011). NetDS: a Cytoscape plugin to analyze the robustness of dynamics and feedforward/feedback loop structures of biological networks. Bioinformatics.

[30] Maslov S, Sneppen K (2002). Specificity and stability in topology of protein networks. Science.

[31] Maslov S, Sneppen K, Alon U (2002). Correlation profiles and motifs in complex networks.

[32] Jeong H, Mason SP, Barabasi AL, Oltvai ZN (2001). Lethality and centrality in protein networks. Nature.

[33] Wuchty S, Stadler PF (2003). Centers of complex networks. J Theor Biol.

[34] Freeman L (1977). A set of measures of centrality based on betweenness. Sociometry.

[35] Shimbel A (1953). Structural parameters of communication networks. Bull Math Biophys.

[36] Bonacich P (1987). Power and centrality: a family of measures. Am J Sociol.

[37] Consortium TU (2015). UniProt: a hub for protein information. Nucleic Acids Res.

[38] Binns D, Dimmer E, Huntley R, Barrell D, O’Donovan C, Apweiler R (2009). QuickGO: a web-based tool for Gene Ontology searching. Bioinformatics.

[39] Estrada E, Rodríguez-Velázquez JA (2005). Subgraph centrality in complex networks. Physical Review E.

[40] Kim H, Anderson R (2012). Temporal node centrality in complex networks. Physical Review E.

[41] Li M, Lu Y, Wang J, Wu FX, Pan Y (2015). A topology potential-based method for identifying essential proteins from PPI networks. IEEE/ACM Trans Comput Biol Bioinform.

[42] Consortium TGO (2008). The Gene Ontology project in 2008. Nucleic Acids Res.

