# Supplementary material for: MORO: a Cytoscape app for relationship analysis between modularity and robustness in large-scale biological networks
Source: BMC Syst Biol. 2016 Dec 23;10(Suppl 4):122. doi: 10.1186/s12918-016-0363-3 (PMC5260057; doi:10.1186/s12918-016-0363-3)
Supplement: Additional file 1: Text S1. — Parallel robustness computation based on the OpenCL. Text S2. Output file by the batch-mode simulation on RBNs. Text S3. Centrality measures. (PDF 753 kb) [file 12918_2016_363_MOESM1_ESM.pdf]

Text S1. Parallel robustness computation based on the OpenCL library

We extended the implementation in a previous study [1] so as to compute in-/out-module robustness. The following figure gives pseudocode describing two important functions, *parallel_computing_attractors_for_all_states* and *parallel_computing_attractors_for_all_rules*, which can compute the attractors in parallel for all initial states (S) and every update rule (F), respectively, given a Boolean network. In computing the attractors, we used an array *ATT* in which each element $ATT[s, f]$ represents an attractor of a network $G(V, A)$ corresponding to the initial state s and the sequence of update rules f. The algorithm iteratively computes state transitions until it arrives at a state that has already been visited. In the figure, dashed blocks denote kernel codes that are executed *in parallel* on CPUs or GPUs. In other words, the new MORO app computes them in parallel by distributing the tested cases to processing elements in the OpenCL device.

```
function[ATT] parallel_computing_attractors_for_all_states(V, A, f, S)
  // V, A: A set of nodes V={v_1, v_2, …, v_N} and a set of links A of a network (Here,
          V[i] represents v_i∈V.)
  // f:   A sequence of update rules (Here, f = f_1 f_2 …f_N and f_i represents the
          update rule with respect to v_i∈V.)
  // S:   A collection of initial states considered for the robustness investigation
          (Here, S[i] represents i^th initial state in S.)
  // ATT: The resulting collection of attractors each of which is represented by a
          sequence of states.

  ATT[0.. 2^|V| -1] ← NULL; // Every element of ATT is initialized by NULL.
  nth[0.. 2^|V| -1] ← 0; // Every element of nth is initialized by 0.

  for i←1 to |S| // for every state
  - - - - - - - - - - - - - - - - - - - - - - - - - - - - - - - - - - -
   s ← S[i];
   if (ATT[s, f] ≠ NULL) continue;
   endif
   traj ← NULL;
   count ← 0;
   while (TRUE)
       count++;
       traj ← traj⊕s; // Here ⊕ represents the string concatenation operation.
       nth[s] ← count;
       s' ← update_states (V, A, f, s); // This computes the next state.
       if (nth[s'] ≠ 0)
          if (ATT[s', f] = NULL)
              att ← traj_{nth[s']..count}; // Given a string t=t_1t_2…t_T, t_{i..j} represents
                                           // t_it_{i+1}…t_{j-1}t_j which is a substring of t.
          else
              att ← ATT[s', f];
          endif
          for j←1 to count
              ATT[traj_j, f] = att;
          endfor
          break;
      else
          s ← s';
      endif
   endwhile
  - - - - - - - - - - - - - - - - - - - - - - - - - - - - - - - - - - -
  endfor
  return ATT;
end
```

```
function [ATT] parallel_computing_attractors_for_all_rules (V, A, F, s)
  // V, A: A set of nodes V={v_1, v_2,…, v_N} and a set of links A of a network
          (Here, V[i] represents v_i∈V.)
  //F:    A collection of sequences of update rules (Here, F[i] represents i^th
          sequence of update rules in F)
  //s:    An initial state considered for the robustness investigation
  //ATT:  The resulting collection of attractors each of which is represented
          by a sequence of states.

  ATT[0..2^|V|-1]←NULL; // Every element in ATT is initialized by NULL.
  for i←1 to |F| // for every rule
  - - - - - - - - - - - - - - - - - - - - - - - - - - - - - - - - - - -
   nth[0.. 2^|V|-1]← 0; // Every element in nth is initialized by 0.
   traj←NULL;
   count = 0;
   while (TRUE)
       count++;
       traj←traj⊕s; // ⊕ represents the string concatenation  operation.
       nth[s] ←count;
       s' ←update_states (V,A, F[i], s); // This computes the next state.
       if(nth[s'] ≠ 0)
           att = traj_{nth[s']..count}; // Given a string t=t_1t_2…t_T, t_{i..j} represents
                                        //t_it_{i+1}…t_{j-1}t_j which is a substring of t.
           ATT[s, F[i]] = att;
           break;
       else
           s←s';
       endif
   endwhile
  - - - - - - - - - - - - - - - - - - - - - - - - - - - - - - - - - - -
  endfor
  returnATT;
end
```

By using *parallel_computing_attractors_for_all_states* and *parallel_computing_attractors_for_all_rules*, we can compute the in-/out-module robustness of a network G against initial-state perturbation and update rule perturbation ($\gamma_{in}(G)$ and $\gamma_{out}(G)$, respectively) as shown in the following pseudocode.

```
function [γin, γout] in-/out-module robustness_initial_state (V, A, f, S. M)
  // V, A: A set of nodes V={v1, v2, …, vN} and a set of links A of a network
           (Here, V[i] represents vi∈V.)
  // f:    A sequence of update rules (Here, f = f1 f2 …fN and fi represents the
           update rule with respect to vi∈V.)
  // S:    A collection of initial states considered for the robustness investigation
           (Here, S[i] represents ith initial state in S.)
  // M:    A set of modules M={m1, m2, …mM) of a network after using module
           detection algorithm. In particular, each node will be belonged to each
           module. In other words, each module contains a number of nodes of a
           network.
  // γin:  The resulting in-module robustness against initial-state perturbations
  // γout: The resulting out-module robustness against initial-state perturbations

  // Step 1: Examine the original attractors.
  ATT ← parallel_computing_attractors_for_all_states (V, A, f, S);

  // Step 2: Examine the changed attractors by initial-state perturbations.
  for i←1 to |S|
        S'[1..|V|] ← NULL; // Every element of S' is initialized by NULL.
        for j←1 to |V|
     s ← S[i];
     sj ← 1- sj; // sj denotes the value of vj in s, and then the resultant s
              // denotes an initial-state perturbation at a node vj∈V.
     S'[j] ← s;
        endfor
    ATT' ← parallel_computing_attractors_for_all_states(V, A, f, S');
  // Step 3: using the Hamming distance measure to examine the similarity be-
  tween four partial attractors (⟨s_in⟩ and ⟨s'_in⟩), (⟨s_out⟩ and s'_out) extracted
  from original attractors and attractor by perturbation based on the modular in-
  formation of each node.
        γin←0;
        γout←0;
        tin←0;     t'in←0;
        tempout←0; temp'out←0;
       for j←1 to |M|
          for k←1 to |Vk|
             tin+=H (⟨s_in⟩, ⟨s'_in⟩);
             tempout+= H (⟨s_out⟩, ⟨s'_out⟩);
          endfor
          t'in+=tin/|Vk|;
          temp'out+=tempout/|Vk|;
       endfor
       γin+=t'in/|M|;
       γout=temp'out/|M|;
     endfor
  γin ←γin/ |S|; // As a result, γin represents the in-module robustness of the giv-
  en network.
  γout ←γout/ |S|; // As a result, γout represents the out-module robustness of the
  given network.
  return γin, γout;
end
```

```
function [γin, γout ] in-/out-module robustness_update_rule (V, A, f, S, M)
  // V, A: A set of nodes V={v1, v2, …, vN} and a set of links A of a network
           (Here, V[i] represents vi∈V.)
  // f:    A sequence of update rules (Here, f = f1 f2 …fN and fi represents the
           update rule with respect to vi∈V.)
  // S:    A collection of initial states considered for the robustness investigation
           (Here, S[i] represents ith initial state in S.)
  // M:    A set of modules M={m1, m2, …mM) of a network after using module
           detection algorithm. In particular, each node will be belonged to each
           module. In other words, each module contains a number of nodes of a
           network.
  // γin:  The resulting in-module robustness against update-rule perturbations
  // γout: The resulting out-module robustness against update-rule perturbations

  // Step 1: Examine the original attractors.
  ATT ← parallel_computing_attractors_for_all_states (V, A, f, S);

  // Step 2: Examine the changed attractors by update-rule perturbations.
  for i←1 to |S|
        F[1..|V|] ← NULL; // Every element of F is initialized by NULL.
        for j←1 to |V|
     f' ← f;
     if (fj = AND ) f'j ← OR;
     else f'j ← AND; // f means an update-rule perturbation at a node vj∈V.
     endif
     F[j] ← f';
        endfor
    ATT' ← parallel_computing_attractors_for_all_rules (V, A, F, S[i]);
  // Step 3: using the Hamming distance measure to examine the similarity be-
  tween four partial attractors (⟨s_in⟩ and ⟨s'_in⟩), (⟨s_out⟩ and s'_out) extracted
  from original attractors and attractor by perturbation based on the modular in-
  formation of each node.
        γin←0;
        γout←0;
        tin←0;     t'in←0;
        tempout←0; temp'out←0;
        for j←1 to |M|
           for k←1 to |Vk|
              tin+=H (⟨s_in⟩, ⟨s'_in⟩);
              tempout+= H (⟨s_out⟩, ⟨s'_out⟩);
           endfor
           t'in+=tin/|Vk|;
           temp'out+=tempout/|Vk|;
        endfor
        γin+=t'in/|M|;
        γout=temp'out/|M|;
      endfor
  γin ←γin/ |S|; // As a result, γin represents the in-module robustness of the giv-
  en network.
  γout ←γout/ |S|; // As a result, γout represents the out-module robustness of the
  given network.
  return γin, γout;
end
```

Text S2. Output file by the batch-mode simulation on RBNs

After the batch-mode simulation is completed, a resultant file "net_based_result.txt" is created, which lists the network-based results. As shown in the figure below, the file consists of 11 results with respect to robustness, modularity and in-/out-module robustness. Each row lists the result of a single RBN.

Column	Name	Description
1	Network ID	The unique identification number of an RBN
2	No.Nodes	The number of nodes of an RBN
3	No.Edges	The number of edges of an RBN
4	sRobustness	The robustness against initial-state perturbation of an RBN
5	rRobustness	The robustness against update-rule perturbation of an RBN
6	No.Modules	The number of modules of an RBN
7	Modularity	The modularity value of an RBN
8	sInModuleR	The in-module robustness against initial-state perturbation of an RBN
9	rInModuleR	The in-module robustness against update-rule perturbation of an RBN
10	sOutModuleR	The out-module robustness against initial-state perturbation of an RBN
11	rOutModuleR	The out-module robustness against update-rule perturbation of an RBN

(Column description in "net_based_result.txt")

Network ID	No.Nodes	No.Edges	sRobustness	rRobustness	No.Modules	Modularity	sInModuleR	rInModuleR	sOutModuleR	rOutModuleR
0	10	9	0.40000000	0.81484375	2	0.38888889	1.00000000	1.00000000	1.00000000	1.00000000
1	10	10	0.70000000	0.88125000	4	0.34000000	1.00000000	1.00000000	1.00000000	1.00000000
2	10	11	0.73906250	0.90000000	2	0.39256198	0.89166665	1.00000000	0.90312505	1.00000000
3	10	12	0.80000000	0.90000000	3	0.30902778	1.00000000	1.00000000	1.00000000	1.00000000
4	10	13	0.80000000	0.70000000	3	0.20118343	1.00000000	1.00000000	1.00000000	1.00000000
5	10	14	0.70000000	0.65625000	4	0.23469388	1.00000000	0.97705078	1.00000000	0.97607422
6	10	15	0.66250000	0.70625000	2	0.21333333	0.94835079	0.98524308	0.94531256	0.99218750
7	10	16	0.70000000	0.67421875	2	0.12500000	1.00000000	0.94372392	1.00000000	0.94443357
8	10	17	0.70000000	0.90000000	3	0.14359862	1.00000000	1.00000000	1.00000000	1.00000000
9	10	18	0.95625000	0.60390625	3	0.20370370	1.00000000	0.94189453	1.00000000	0.94091797
10	10	19	0.50000000	0.64531250	2	0.12188366	1.00000000	1.00000000	1.00000000	1.00000000
11	10	20	0.90000000	0.60468750	4	0.21125000	1.00000000	0.88037109	1.00000000	0.87719727
12	10	21	1.00000000	0.40000000	2	0.25396825	1.00000000	0.74945736	1.00000000	0.74207890
13	10	22	1.00000000	0.80000000	2	0.13119835	1.00000000	1.00000000	1.00000000	1.00000000
14	10	23	1.00000000	0.60000000	2	0.15122873	1.00000000	0.93437499	1.00000000	0.93500000
15	10	24	0.91718750	0.40859375	3	0.15277778	0.94357634	0.70572895	0.95703125	0.61111122
16	10	25	0.79609375	0.30000000	3	0.16080000	0.87532550	0.93164062	0.87011719	0.93554688

(Example of "net_based_result.txt")

Text S3. Centrality measures

In this study, centrality measures are used to measure how much a module is central. We provide five centrality measures: degree, closeness, betweenness, stress, and eigenvector.

- *Degree* (DEG) indicates the ability to communicate directly with other nodes [2].

- *Betweenness (BEW)* quantifies the ability of a node to monitor communication between other nodes through the shortest path [3]. More specifically, it is defined as follows:

$$BEW(v) = \sum_{u \neq w \in V \setminus \{v\}} \frac{\sigma_{uw}(v)}{\sigma_{uw}},$$

  where σ_{uw} denotes the number of shortest paths between u and w, and $\sigma_{uw}(v)$ denotes the one that v passes through.

- *Stress (STR)* is based on the enumeration of shortest paths [4], and is similar to betweenness; however, instead of the sum of the relative number of shortest paths for each pair of nodes, stress is the sum of the absolute number of shortest paths. This gives an approximation of the amount of 'work' or 'stress' the protein has to sustain in the network:

$$STR(v) = \sum_{u \neq w \in V \setminus \{v\}} \sigma_{uw}(v).$$

- *Closeness (CLO)* measure uses the sum of the minimal distances from a node to all other nodes [5]. The closeness measure is defined as the reciprocal of this sum:

$$CLO(v) = \frac{1}{\sum_{u \in V \setminus \{v\}} dist(v,u)}.$$

- *Eigenvector (EIG)* measure is defined as the principal eigenvector of the adjacency matrix, D, of the network [6]. It simulates a mechanism in which each node affects all of its neighbors simultaneously. Given the adjacency matrix D, the eigenvector (e) and eigenvalue (Λ) are obtained by using the equation $\Lambda e = De$. Let e_1 be the eigenvector corresponding to the largest (principal) eigenvalue. Then, the eigenvector-based centrality of a node can be denoted by the corresponding component of e_1:

$$EIG(v) = e_1(v).$$

References

1. Trinh H-C, Le D-H, Kwon Y-K (2014) PANET: A GPU-Based Tool for Fast Parallel Analysis of Robustness Dynamics and Feed-Forward/Feedback Loop Structures in Large-Scale Biological Networks. PLoS ONE 9: e103010.
2. Jeong H, Mason SP, Barabasi AL, Oltvai ZN (2001) Lethality and centrality in protein networks. Nature 411: 41-42.
3. Freeman L (1977) A Set of Measures of Centrality Based on Betweenness. Sociometry 40: 35-41.
4. Shimbel A (1953) Structural parameters of communication networks. Bulletin of Mathematical Biology 15: 501-507.
5. Wuchty S, Stadler PF (2003) Centers of complex networks. Journal of Theoretical Biology 223: 45-53.
6. Bonacich P (1987) Power and Centrality: A Family of Measures. American Journal of Sociology 92: 1170-1182.
