# Supplementary material for: MORO: a Cytoscape app for relationship analysis between modularity and robustness in large-scale biological networks
Source: BMC Syst Biol. 2016 Dec 23;10(Suppl 4):122. doi: 10.1186/s12918-016-0363-3 (PMC5260057; doi:10.1186/s12918-016-0363-3)
Supplement: Additional file 2: Figure S1. — Analysis results of the HSN network by MORO. Figure S2. Correlations between the modularity and robustness of 6,400 random Boolean networks where the number of nodes is 50 and the number of interactions is in the range of [49, 2031]. Figure S3. Changes of module centrality values against the module size in the HSN network. Figure S4. Changes of module centrality values against the module size in STKE-shuffled random networks. Figure S5. Changes of module centrality values against the module size in HSN-shuffled random networks. Figure S6. Correlation between module centrality values and in-/out-module robustness in the STKE network. Figure S7. Correlation between module centrality values and in/out-module robustness in the HSN network. (PDF 1052 kb) [file 12918_2016_363_MOESM2_ESM.pdf]

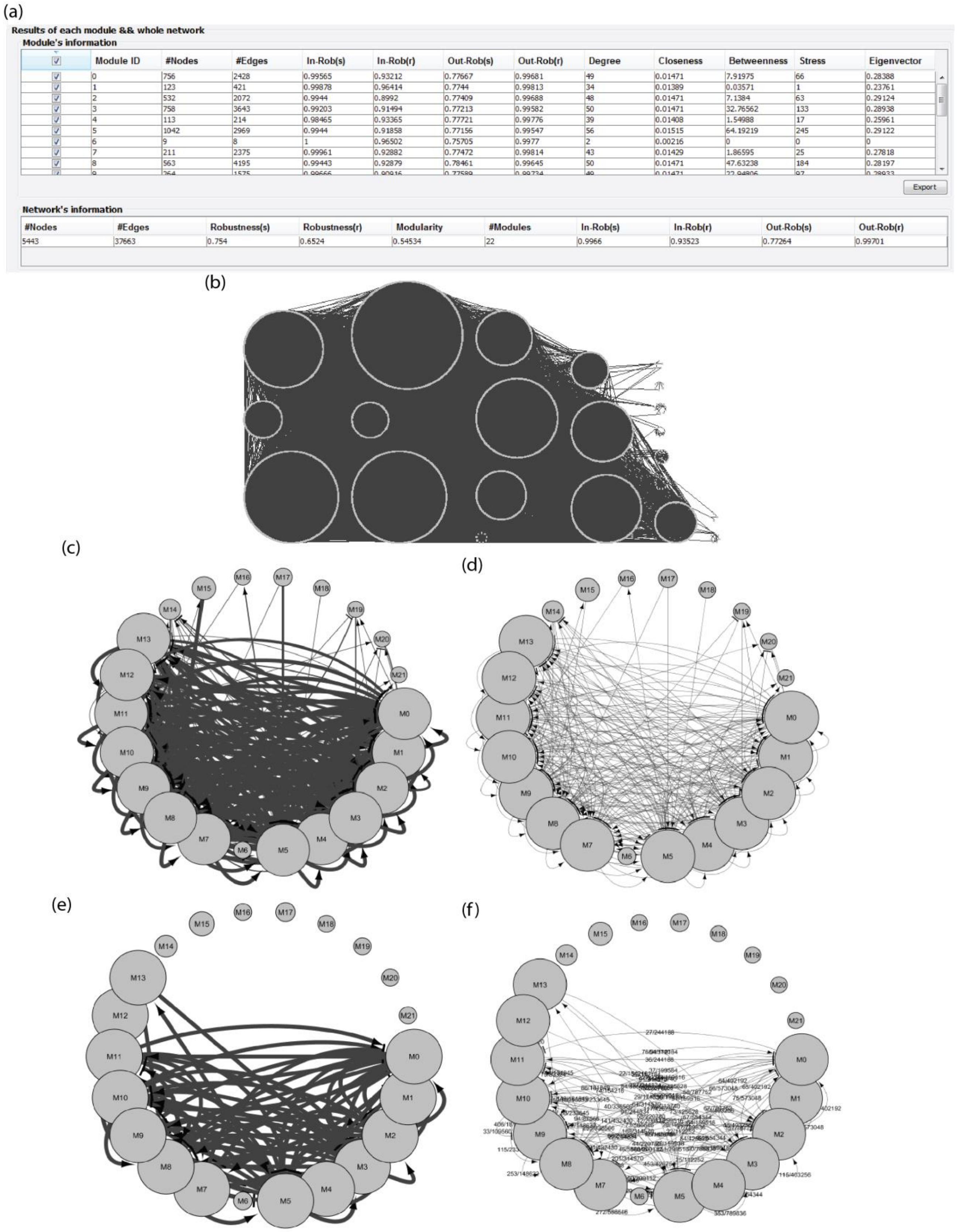

Results of each module && whole network

Module's information

☑	Module ID	#Nodes	#Edges	In-Rob(s)	In-Rob(r)	Out-Rob(s)	Out-Rob(r)	Degree	Closeness	Betweenness	Stress	Eigenvector
☑	0	756	2428	0.99565	0.93212	0.77667	0.99681	49	0.01471	7.91975	66	0.28388
☑	1	123	421	0.99878	0.96414	0.7744	0.99813	34	0.01389	0.03571	1	0.23761
☑	2	532	2072	0.9944	0.8992	0.77409	0.99688	48	0.01471	7.1384	63	0.29124
☑	3	758	3643	0.99203	0.91494	0.77213	0.99582	50	0.01471	32.76562	133	0.28938
☑	4	113	214	0.98465	0.93365	0.77721	0.99776	39	0.01408	1.54988	17	0.25961
☑	5	1042	2969	0.9944	0.91858	0.77156	0.99547	56	0.01515	64.19219	245	0.29122
☑	6	9	8	1	0.96502	0.75705	0.9977	2	0.00216	0	0	0
☑	7	211	2375	0.99961	0.92882	0.77472	0.99814	43	0.01429	1.86595	25	0.27818
☑	8	563	4195	0.99443	0.92879	0.78461	0.99645	50	0.01471	47.63238	184	0.28197
☑	9	264	1575	0.99666	0.90916	0.77589	0.99734	49	0.01471	22.94806	97	0.28933

Export

Network's information

#Nodes	#Edges	Robustness(s)	Robustness(r)	Modularity	#Modules	In-Rob(s)	In-Rob(r)	Out-Rob(s)	Out-Rob(r)
5443	37663	0.754	0.6524	0.54534	22	0.9966	0.93523	0.77264	0.99701

Figure S1. Analysis results of the HSN network by MORO. **(a)** A summary table. Modularity and robustness results in module and network levels are listed in the upper and the lower tables, respectively. **(b)** Result of the detailed visualization mode. We found a total of 22 modules each of which is represented by a circular list of genes. **(c)-(d)** Results of the brief visualization mode with absolute and relative relations, respectively. Each module is represented by a single group node whose radius is proportional to the number of nodes belonging to the module. The weight of a link denotes the number of interactions between the corresponding pair of modules and the ratio of the number of interactions between a pair of modules to the maximal possible number of interactions between them in (c) and (d), respectively. **(e)-(f)** The reduced visualization results. They are subnetworks induced from (c) and (d), respectively, by removing all links except about 30% of links with the highest weight values (This is performed by specifying the appearance ratio parameter in MORO).

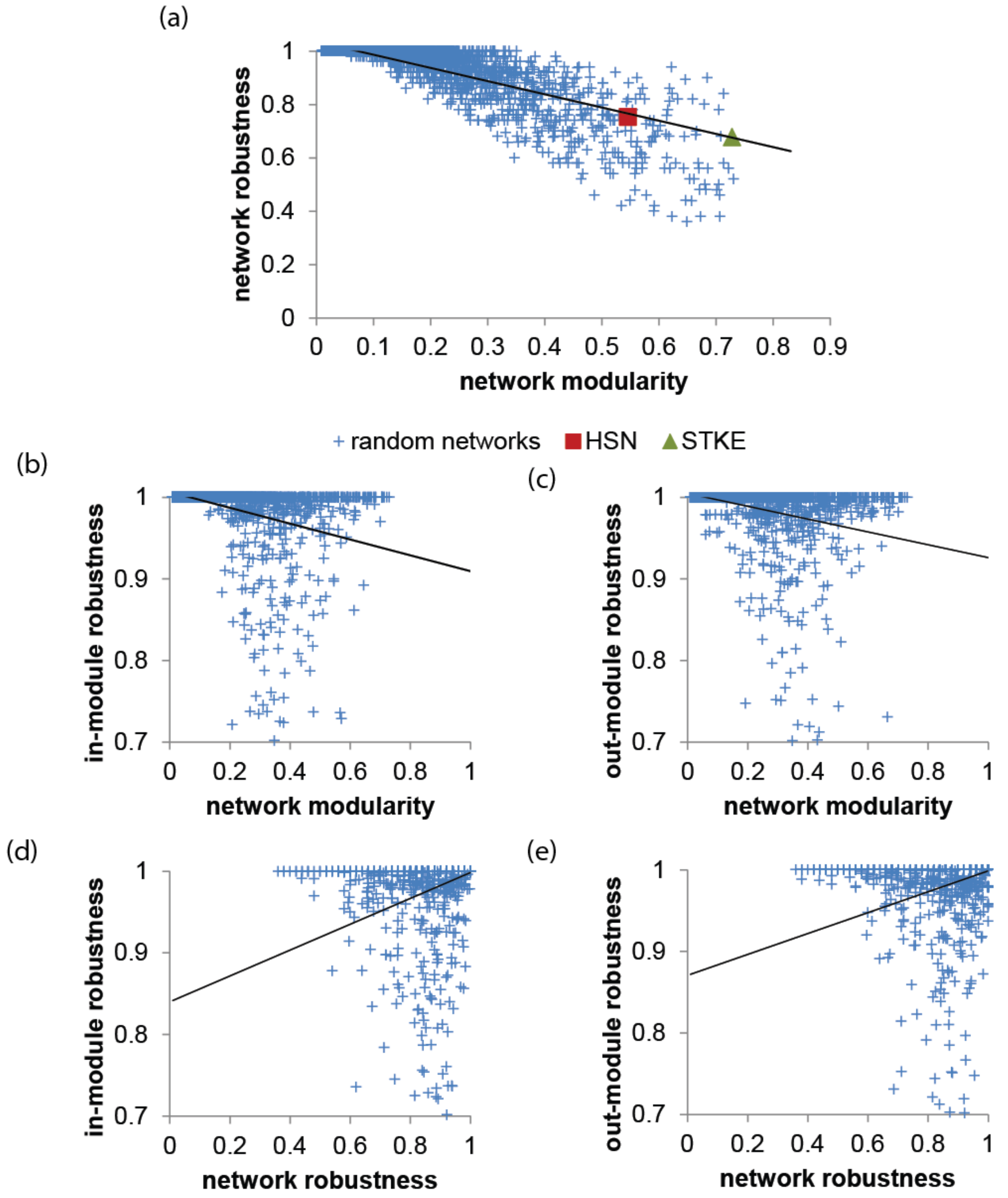

Figure S2. Correlations between the modularity and robustness of 6,400 random Boolean networks where the number of nodes is 50 and the number of interactions is in the range of [49, 2031]. **(a)** Relationship between network modularity and robustness: the correlation coefficient was negative (correlation coefficient = −0.80303 with p-value < 10^{-4}). The results for HSN and STKE, denoted by the rectangular and triangular points, respectively, were very close to the linear regression line. **(b)** Relationship of the network modularity to the in-module robustness (correlation coefficient = −0.30383 with p-value <10^{-4}). **(c)** Relationship between network modularity and out-module robustness (not significant). **(d)** Relationship of the network robustness to the in-module robustness (correlation coefficient = 0.27801 with p-value <10^{-4}). **(e)** Relationship between network robustness and out-module robustness (not significant).

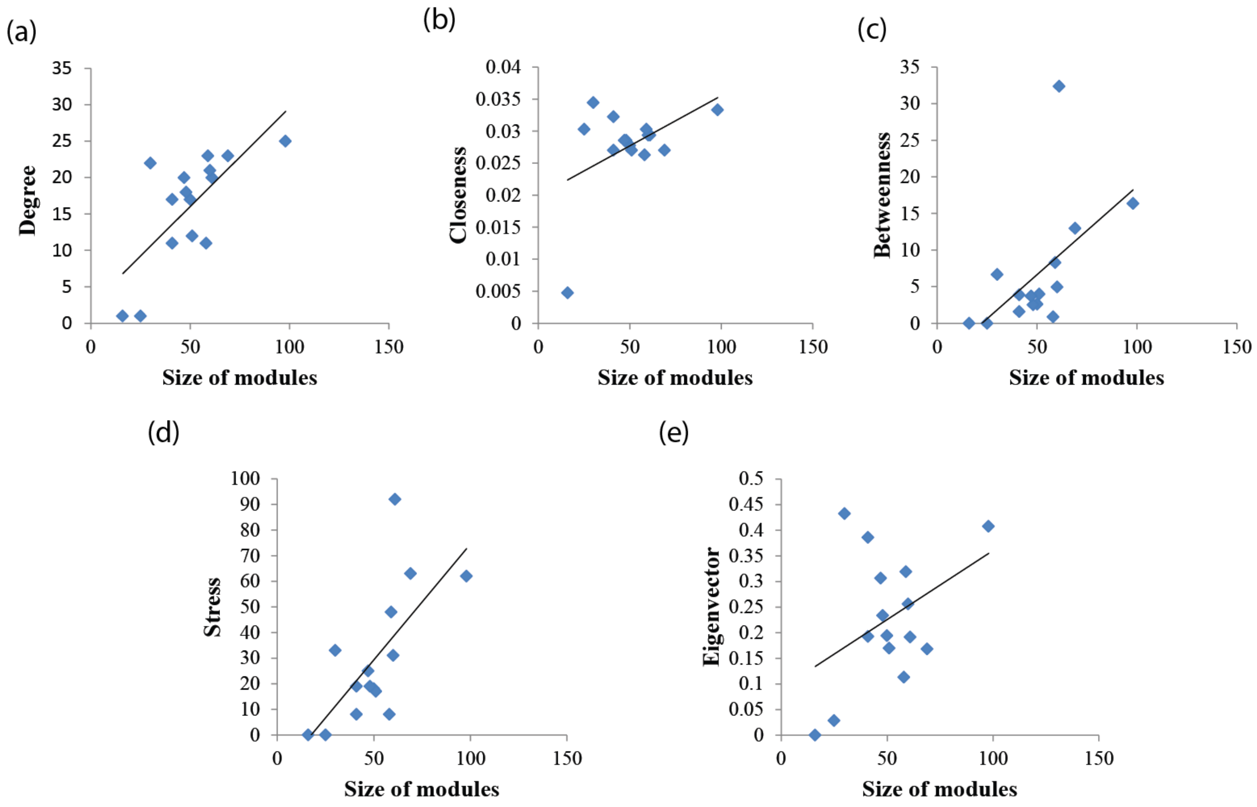

Figure S3. Changes of module centrality values against the module size in the HSN network. **(a)-(e)** Results with respect to degree, closeness, betweenness, stress, and eigenvector. The module size which is defined as the number of nodes belonging to the module showed positive relationships with all module centrality measures except closeness. The correlation coefficients in (a), (c), (d), and (e) were 0.79367, 0.599553, 0.70063, and 0.870837, respectively, with all p-value $< 10^{-4}$.

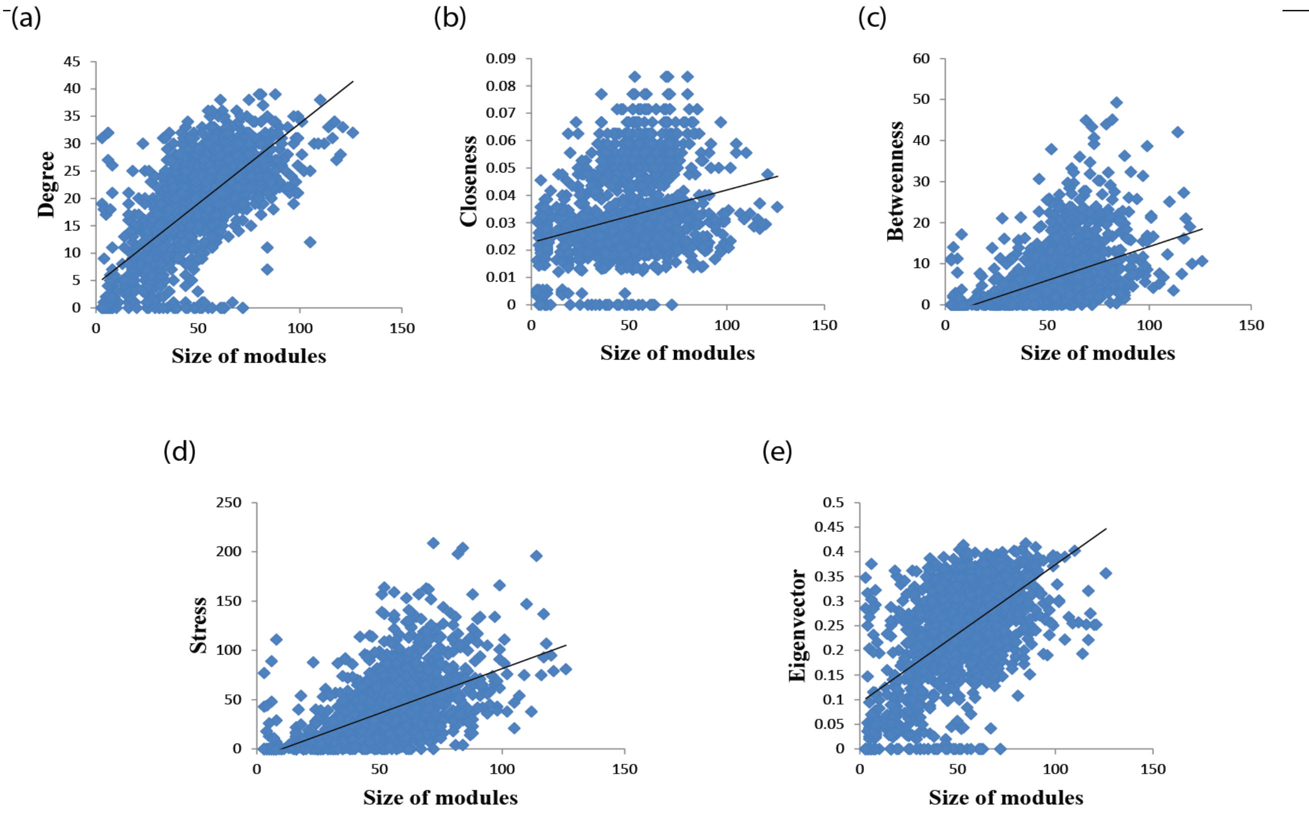

Figure S4. Changes of module centrality values against the module size in STKE-shuffled random networks. We generated 100 random networks by shuffling interactions of the STKE network while preserving a degree distribution. **(a)-(e)** Results with respect to degree, closeness, betweenness, stress, and eigenvector. The module size which is defined as the number of nodes belonging to the module showed positive relationships with all module centrality measures. The correlation coefficients in (a), (b), (c), (d), and (e) were 0.70475, 0.26639, 0.50143, 0.57625, and 0.58761, respectively, with all p-value < 10^{-4}.

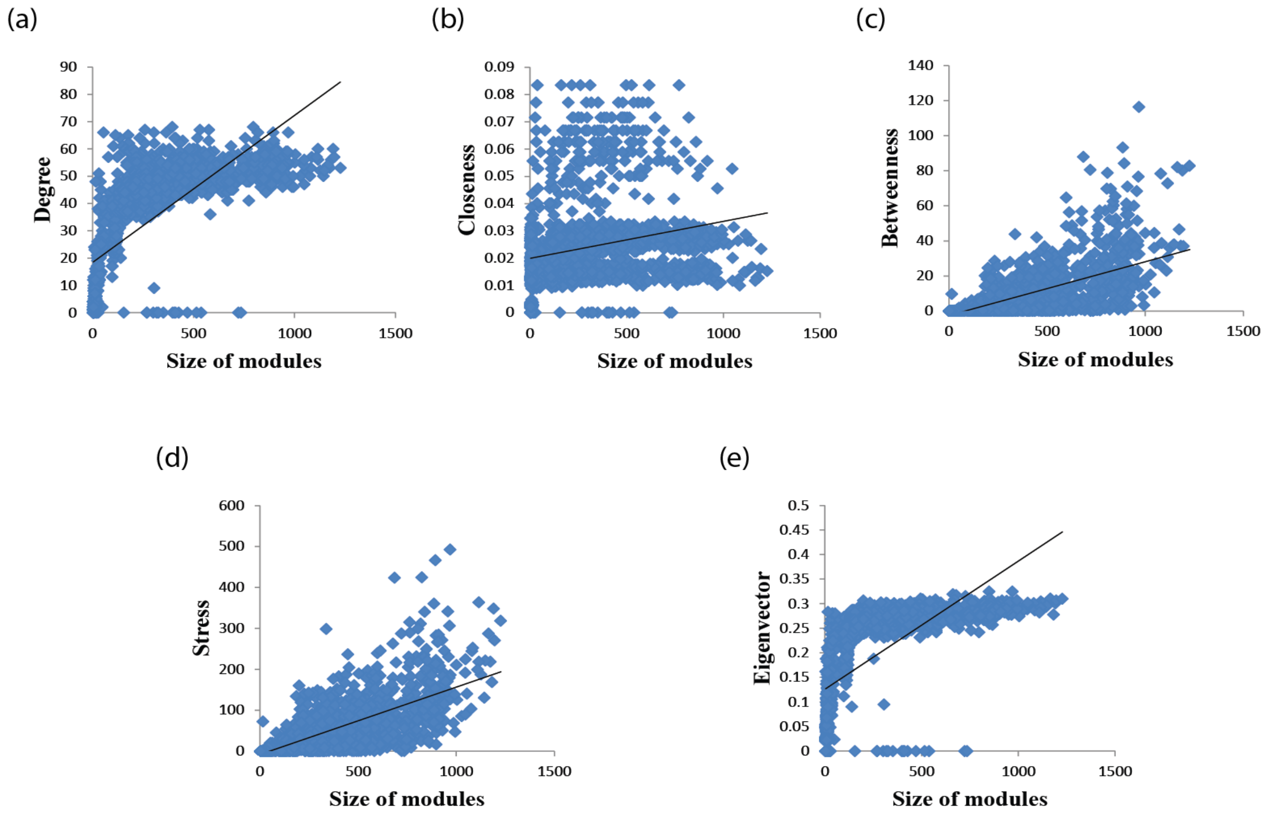

Figure S5. Changes of module centrality values against the module size in HSN-shuffled random networks. We generated 100 random networks by shuffling interactions of the HSN network while preserving a degree distribution. **(a)-(e)** Results with respect to degree, closeness, betweenness, stress, and eigenvector. The module size which is defined as the number of nodes belonging to the module showed positive relationships with all module centrality measures. The correlation coefficients in (a), (b), (c), (d), and (e) were 0.73027, 0.24344, 0.66850, 0.74306, and 0.67059, respectively, with all p-value $< 10^{-4}$.

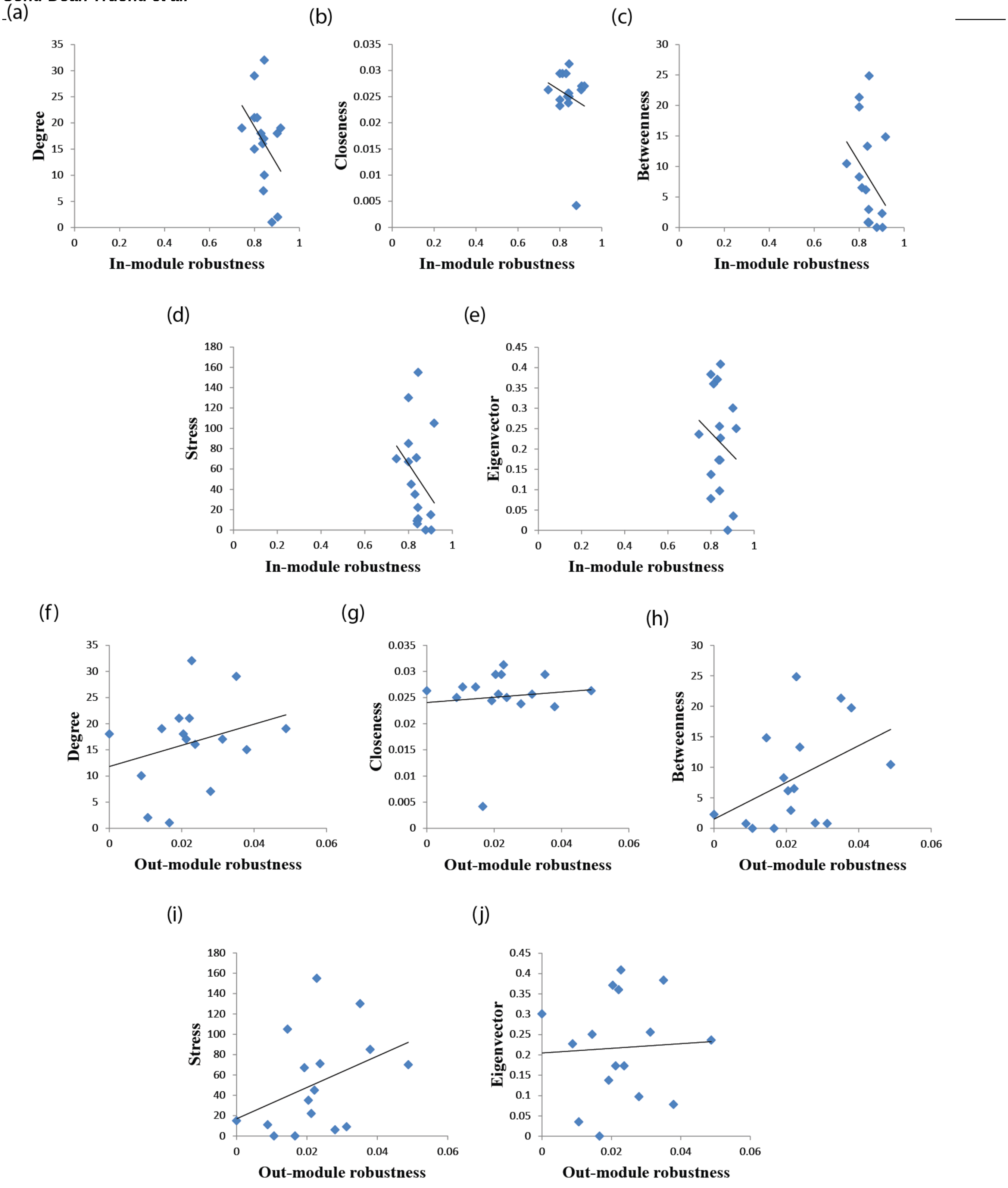

Figure S6. Correlation between module centrality values and in-/out-module robustness in the STKE network. **(a)-(e)** Correlations of in-module robustness with degree, closeness, betweenness, stress, and eigenvector, respectively. There was no significant relation (all p-values > 0.13279). **(f)-(j)** Correlations of out-module robustness with degree, closeness, betweenness, stress, and eigenvector, respectively. There was no significant relation (all p-values > 0.09143).

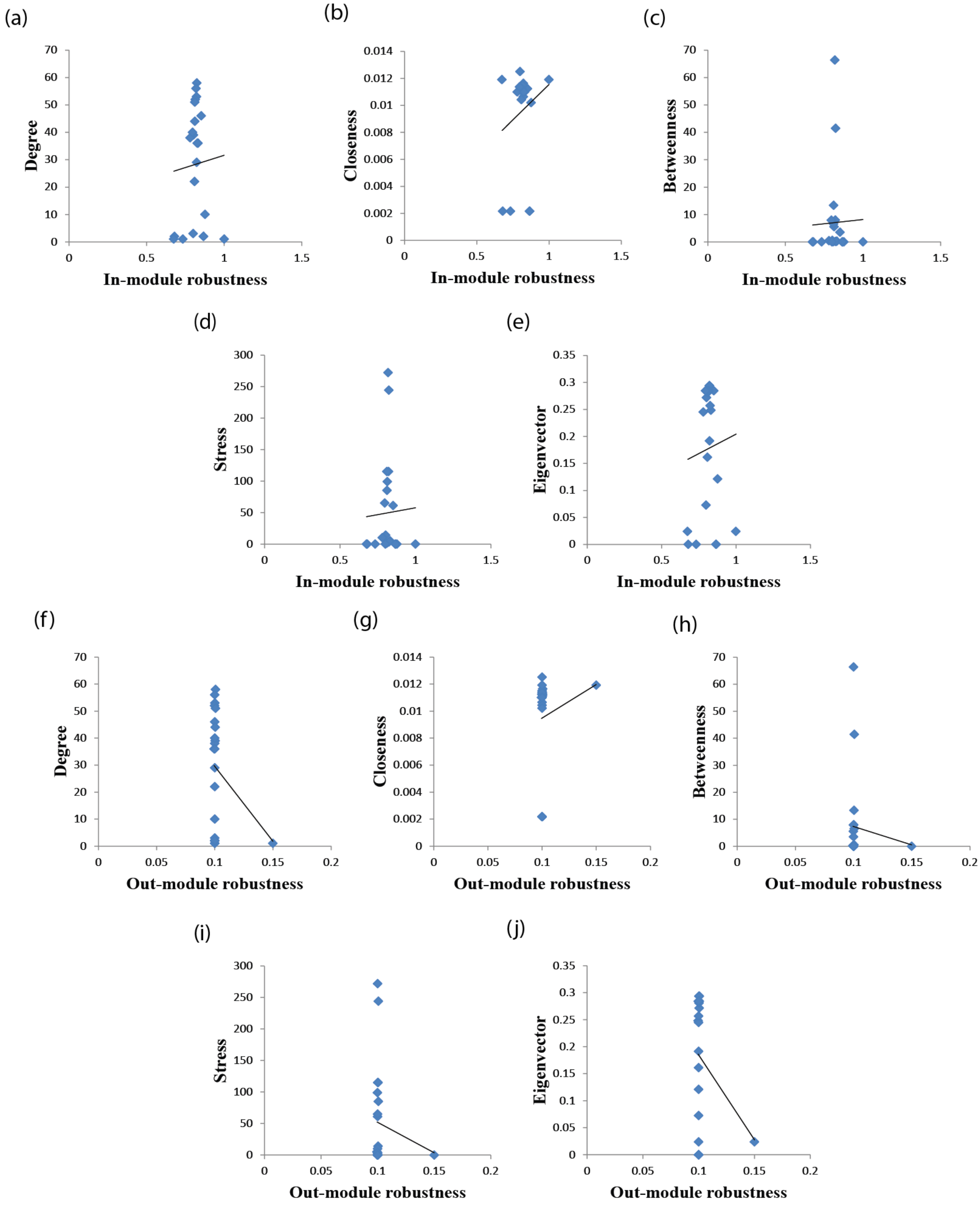

Figure S7. Correlation between module centrality values and in/out-module robustness in the HSN network. **(a)-(e)** Correlations of in-module robustness with degree, closeness, betweenness, stress, and eigenvector, respectively. There was no significant relation (all p-values > 0.39269). **(f)-(j)** Correlations of out-module robustness with degree, closeness, betweenness, stress, and eigenvector, respectively. There was no significant relation (all p-values > 0.21193).
